# The Flexner Report of 1910 and Its Impact on Complementary and Alternative Medicine and Psychiatry in North America in the 20th Century

**DOI:** 10.1155/2012/647896

**Published:** 2012-12-26

**Authors:** Frank W. Stahnisch, Marja Verhoef

**Affiliations:** Department of Community Health Sciences, Faculty of Medicine, University of Calgary, Teaching Research and Wellness Building, 3E41, 3280 Hospital Drive N.W., Calgary, AB, Canada T2N 4Z6

## Abstract

America experienced a genuinely vast development of biomedical science in the early decades of the twentieth century, which in turn impacted the community of academic psychiatry and changed the way in which clinical and basic research approaches in psychiatry were conceptualized. This development was largely based on the restructuring of research universities in both of the USA and Canada following the influential report of Johns Hopkins-trained science administrator and politician Abraham Flexner (1866–1959). Flexner's report written in commission for the Carnegie Foundation for the Advancement of Teaching in Washington, DC, also had a major influence on complementary and alternative medicine (CAM) in psychiatry throughout the 20th century. This paper explores the lasting impact of Flexner's research published on modern medicine and particularly on what he interpreted as the various forms of health care and psychiatric treatment that appeared to compete with the paradigm of biomedicine. We will particularly draw attention to the serious effects of the closing of so many CAM-oriented hospitals, colleges, and medical teaching programs following to the publication of the Flexner Report in 1910.

## 1. Introduction

**Figure 1:**
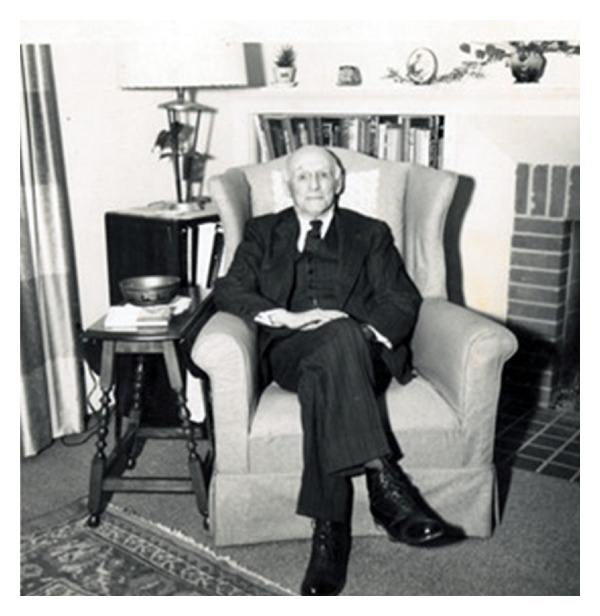
Abraham Flexner (1866–1959).

Between 1900 and 1930, the United States of America and Canada witnessed a major expansion of research activities in the field of biomedicine (most notably impacting academic psychiatry, clinical research in internal medicine, and the integration of laboratory-based pathology), a process which became strongly connected with the great and lasting transformation of modern universities, colleges, and hospitals [1]. This development was at the same time flanked by an influential strategic report, which US science administrator and politician Abraham Flexner (1866–1959) had written in 1909, subsequently published by the Carnegie Foundation for the Advancement of Teaching in 1910 [2]. Flexner himself (Figure 1) was trained in the natural sciences at the preeminent Johns Hopkins University in Baltimore, MD (USA), where he received a German-style, research education which was grounded in intensive laboratory work and the active pursuit of scientific experimentation on both graduate and undergraduate levels. Since its inception by founding dean William Henry Welsh (1850–1934), in 1884, the medical school had focused on bedside teaching, concise, and standardized clinical observations and the early introduction of laboratory experimentation and research work. This science-based form of academic education had a lasting effect on Flexner's views about the status of modern medicine, who incessantly promoted this new scientific paradigm of medical education and research. To him, illegitimate “nonscientific” approaches in the medical marketplace (such as the offerings of folk psychologists, naturopaths, homoeopaths, chiropractors, and osteopaths) were actively competing with the scientific paradigm of research and education represented at major American and Canadian universities at the time [3].

At the bottom of these events lays a superb growth in the state funding for biomedical research, new psychiatric hospitals, and asylums, along with increasing health care support through company-based plans and state welfare insurance corporations emerging in the “American Progressive Era” since the 1890s [4]. These initiatives also included additional monetary support for biomedical research and medical education, and they were made possible by philanthropic foundations such as the Rockefeller Foundation and the Carnegie Foundation for the Advancement of Teaching in New York City. American medical schools and academic psychiatric departments—most prominently represented in the Clinical Department of Psychiatry headed by the Swiss émigré psychiatrist Adolph Meyer (1866–1950)—benefitted greatly from the renewed and increased financial support from external sources after the end of WWI, when the number of scientific research publications reached an unprecedented level and for the first time compared favorably with former leading countries, such as France, Germany, and Britain [5, 6]. Flexner's report on “Medical Education in the United States and Canada” was written in the middle of the bourgeoning economic and social context following the turn of the century, and it exerted a significant impact on the growth of North American biomedicine, yet it also had a large deleterious effect on the later development of complementary and alternative medicine (CAM) in psychiatry during the 20th Century. Mediated through the commissions of the Carnegie Foundation for the Advancement of Teaching and its Carnegie Foundation Washington, D C Office, Flexner's Report subsequently led to shutting down the majority of CAM-oriented colleges and programs (e.g., medical schools, homoeopathic colleges, and some psychiatric institutions) before and after WWI [7].

Summarizing the context in which Flexner's report appeared, modern scientific medicine—as it had emerged particularly with the French experimental physiologists in the 19th Century—[8] had come to be challenged by a variety of competing contemporary approaches within the medical marketplace (such as naturopathy, traditional homoeopathy, chiropractic, osteopathic medicine, and eclectic forms of therapy) [9]. And while having himself been trained in the scientific paradigm at Johns Hopkins University, Flexner developed a great reservation against the reliability and value of other “nonconformist” approaches in medicine and psychiatry which he pejoratively attacked as “charlatanism” and “quackery,” wanting to weed them out from the modern canon of North American medicine [10]. Flexner became adamant in his strive and polemics against all training facilities that offered education and postgraduate work in the above-mentioned fields and advocated for the closing of nearly eighty percent of all the contemporary programs in homeopathy, naturopathy, eclectic therapy, physical therapy, osteopathy, and chiropractic. He had listed these programs in his report under the pejorative titles of the “medical sects” and stated that he openly aimed to “antagonize” them through the publication of his report, since he saw no firm juridical way to discard these nonbiomedical approaches on the American medical and psychiatric market. Only very few institutions (approximately twenty percent of those mentioned in the Flexner Report) were subsequently able to comply with Flexner's constraints and prescriptions, while most had to shut their doors forever, particularly those in the already medically underserved large rural areas of the American Midwest and the Southern States [11].

In this paper, we will begin by outlining some of the basic assumptions of Abraham Flexner's report to the Carnegie Foundation and its continuing effects on the North American clinical and research landscape in CAM and psychiatry. We then explore some of the antagonisms between the “biomedical model” of health research and nonconventional approaches that Flexner had subsumed under the “medical sects” of the time (e.g., homoeopathy, naturopathy, and homoeopathy, etc.), while pointing to the schism in medicine that Flexner had introduced and further aggravated and which the Canadian medical historian Don G. Bates (1940–2001) has so intriguingly explored and analysed as follows:

*Recently, and for slightly different reasons, this unusual modern, scientific form of medicine [as it had developed during the 19th century] has also given rise to another term: biomedicine. The bio, of course, is meant to point to its strong biological and therefore material and scientific orientation, but the term is frequently used in a critical, even mildly pejorative sense, in order to emphasize the ways in which this caricature fails to make adequate provision for the social and cultural complexities that form part of any medical practice […]. [12].*

In the final part of our paper, we look at the more specific aftereffects of the Flexner Report on North American medicine and psychiatry, while keeping in mind that nearly fifty percent of CAM-treated patients today are suffering from psychiatric disorders and symptoms [13]—including, for example, anxiety disorders, depression, bipolar, and personality disorders—not rarely treated in conjunction with traditional psychiatric approaches from biological psychiatry, psychoanalysis, and behavioural therapies.

## 2. Methods

**Figure 2:**
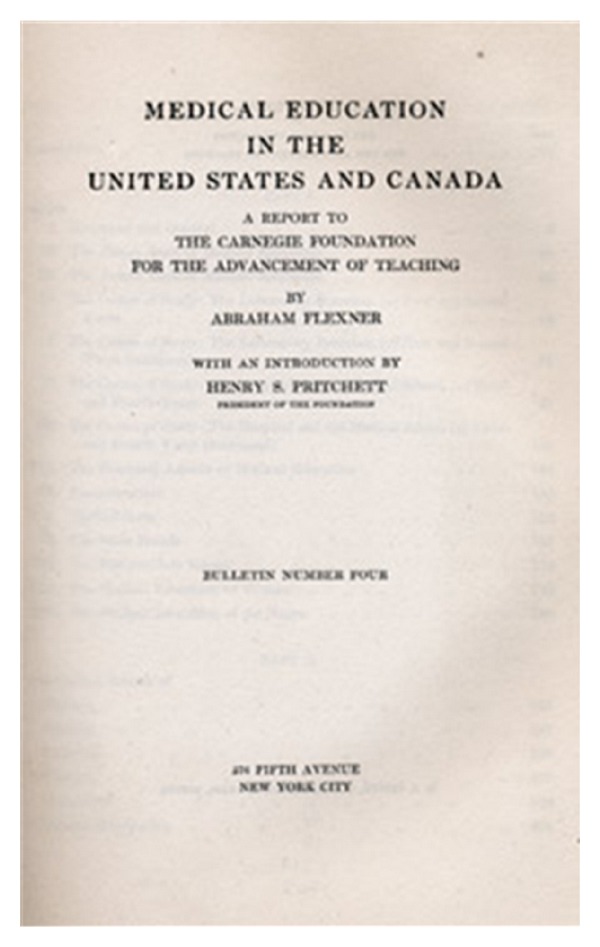
Front page of the Flexner Report of 1910.

Our historiographical research in this paper is based on an analysis of Flexner's *Medical Education in the United States and Canada: A Report to the Carnegie Foundation for the Advancement of Teaching* (1910) (Figure 2) and the available secondary scholarly, medical, and psychiatric literature on the subject. By way of an introduction, textbooks and journal articles on Complementary and Alternative Medicine and Psychiatry are also briefly discussed. Finally, we will scrutinize gray literature and pamphlets published by both the American National Institutes of Health (NIH) and the Canadian Institutes of Health Research (CIHR), as these pertained to the relationship between the biomedical paradigm and CAM-related approaches after the publication of the Flexner Report and, in particular, the inclusion of complementary and alternative therapies and approaches in psychiatry during the second half of the 20th century. This perspective will allow the impact of the Flexner Report to be placed within a contemporary context and its long-lasting effects analyzed.

## 3. Results

### 3.1. The Period Ensuing from the Flexner Report from 1910s to 1940s

The decades following the publication of the Flexner Report witnessed considerable pressure on all nontraditional forms of medical and health care training, which would nowadays be associated with CAM, as “a group of diverse medical and health care systems, practices, and products that are not presently considered to be part of conventional medicine” [14]. In his report, Flexner had made the following claims about the new “standardization” of American medical education:

*Scientific medicine therefore brushes aside all historic dogma. It gets down to details immediately. No man is asked in whose name he comes—whether that of [Samuel] Hahnemann [1755–1843], [Benjamin] Rush [1746–1813], or of some more recent prophet. But all are required to undergo rigorous cross-examination. […] There is no need, just as there is no logical justification, for the invocation of names or creeds, for the segregation from the larger body of established truth of any particular set of truths or supposed truths as especially precious. […] The tendency to build a system out of a few partially apprehended facts, deductive inference filling in the rest, has not indeed been limited to medicine, but it has nowhere had more calamitous consequences [...]. (The original text can be found in: Flexner, 1910 *[2]). **

Rejecting historical forms of knowledge because of their traditional renown and medical educators' authority—including that of the acclaimed “father of American psychiatry” and, signatory of the Declaration of Independence, Benjamin Rush, who worked at the first academic hospital in Pennsylvania and who wrote a pioneering American textbook on mental disease, entitled *Observations and Inquiries upon the Diseases of the Mind *(1812)—was a major part of Flexner's general criticisms of contemporary medical programs. In particular, he dispensed with the continued use of bloodletting, leeches, and purging, as advocated for by Rush, in American psychiatric wards throughout the 19th and the early years of the 20th century. Flexner especially disapproved that such treatments were experimentally unproven nor statistically assessed. Following to his reasoning, these treatments did not adhere to the “gold standard” of modern medical education in biomedicine, that is, the laboratory-based and bedside-oriented Johns Hopkins model of medical research. He particularly criticized that many of the teaching programs in the traditional medical colleges and psychiatric hospitals had no experimental physiological, experimental physiological laboratories, calling them “filthy” and “unhygienic” institutions [2]. His rhetoric would of course stir massive public criticisms in North America, at the time, when rather more than less medical and psychiatric care facilities and training programs were needed, especially in the underserved states of the American Midwest and South and the Canadian Atlantic and Prairie Provinces [15]:

*Of complete [M.D. granting] homeopathic schools, Boston University, the New York Homeopathic College, and the Hahnemann of Philadelphia alone possess the equipment necessary for the effective routine teaching of the fundamental branches. […] Of the remaining homeopathic schools, four are weak and uneven: the Hahnemann of San Francisco and the Hahnemann of Chicago have small, but not altogether inadequate, equipment for the teaching of chemistry, elementary pathology and bacteriology; the Cleveland school offers an active course in experimental physiology. Beyond ordinary dissection and elementary chemistry, they offer little else. […] Six schools remain—all utterly hopeless: [Hering-Chicago, Southwestern, Cincinnati, Atlantic-Baltimore, Detroit & Kansas City]. The buildings are filthy and neglected. At Louisville no branch is properly equipped; in one room, the outfit is limited to a dirty and tattered manikin; in another, a single guinea pig awaits his fate in a cage. (The original text can be found in: Flexner, 1910 *[2]).**

By also alluding to medical luminary of Sir William Osler [1849–1919] and the latter's preceding criticisms of homoeopathy, Flexner integrated a local aim with a general political one in order to promote modern biomedical and reductionist strategies in medical and psychiatric education. Canadian icon of medicine, the internist and pathologist William Osler belonged to the founding fathers of the Johns Hopkins University Medical School—together with the American pathologist William H. Welch, the gynaecologist Howard Kelly (1858–1943), and the surgeon William Stewart Halsted (1852–1922). Their program for restructuring American medical education was likewise based on the modern natural sciences, which aligned well with Flexner's strategy and Johns Hopkins' strive for preeminence among major American medical schools [16]:

*Logically, no other outcome is possible. The ebbing vitality of homeopathic [medical] schools is a striking demonstration of the incompatibility of science and dogma. […] Science, once embraced, will conquer the whole. Homeopathy has two options: one to withdraw into the isolation in which alone any peculiar tenet can maintain itself; the other to put that tenet into the melting-pot. Historically it undoubtedly played an important part in discrediting empirical allopathy. But laboratories of physiology and pharmacology are now doing that work far more effectively than homeopathy; and they are at the same time performing a constructive task for which homeopathy, as such, is unfitted. It will be clear, then, why, when outlining a system of schools for the training of physicians on scientific lines, no specific provision is made for homeopathy. […] “A new school of [medical] practitioners has arisen,” says Dr. [William] Osler, “which cares nothing for homeopathy [...]. (The original text can be found in: Flexner, 1910 *[2]).”**

**Figure 3:**
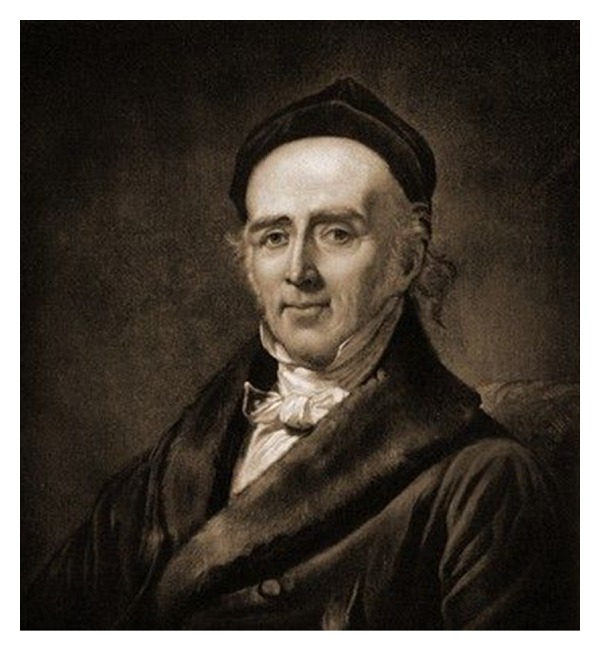
Samuel Hahnemann (1755–1843).

**Figure 4:**
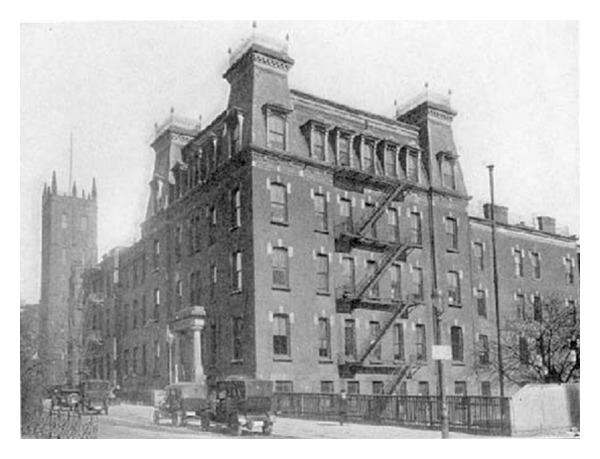
Community health provisions through homoeopathic neighborhood and district hospitals.

The process of introducing graduate schools for the purpose of scientific research—following the example of the German universities during the latter half of the 19th century—would also change the hierarchies in medicine, since science-based faculties claimed themselves that they had a better understanding of pathophysiology, pharmacology, and treatment options than any other institutions. This even included leading traditional medical colleges, such as some of the oldest homeopathic schools, for example, The Pennsylvania Hospital in Philadelphia and Palmer's Chiropractic School in Davenport, NH, USA. They had been established on pre-18th century styles of medical education—inaugurated, for example, in the spirit of Samuel Hahnemann (Figure 3)—and were primarily patient centered, often humanistically oriented and aligned with community medicine and mental health perspectives (Figure 4) [17].

It is not a difficult task to determine how Flexner's observations and criticisms came to influence the development of Complementary and Alternative Medicine and Psychiatry in North America, since this process can be described as a major hindrance for the field to develop further. Among the recommendations of the Flexner Report were, for example, that the admission to a medical school should require, at minimum, a high school diploma and at least two years of college or university study, primarily devoted to basic science. The length of medical education was estimated to be four years, on top of basic science education and primary college graduation, a requirement which the Committee on Continuous Medical Education (CME) of the American Medical Association (AMA) had already agreed upon in 1905.

Furthermore, medical schools should be part of larger research universities, since a proper stand-alone medical school would have to charge fees that were too high for both of its patients and the students in its educational programs and thus would not allow the school to break even. In addition, Flexner envisioned clinical teaching in academically oriented hospitals, where thoughtful physicians and psychiatrists would pursue research stimulated by the questions that arose in the course of patient care and teach their students to do the same. In general, the report triggered a much-needed reform in the standards, organization, and curriculum of North American medical schools and also resulted in a strong emphasis on formal analytic reasoning and positivism in medical science.

A mediating position, one could argue, was taken by the Swiss-American psychiatrist Adolph Meyer, who, as mentioned above, directed the most influential clinical department of psychiatry in North America for more than forty years, and as a clinical professor at Johns Hopkins' School of Medicine, he balanced the Flexnerian demands for rigorous laboratory-based training in medicine with certain nonreductionist views inherent to psychiatry and mental health care. In fact, part of Meyer's academic success and full acceptance in the psychiatric community in the USA and Canada was in line with his reception at Johns Hopkins University of the thorough research program that Emil Kraeplin (1856–1926) had developed at the Clinical Department of Psychiatry at the University of Munich in 1910, while likewise promoting psychohygiene and facilitating the development of psychosomatic medicine—which for Meyer, similar to Flexner, was also a form of following the academic example of the German-speaking universities [18]. Despite the important role ascribed to the Flexner Report, for example, the increase of medical professionalism, closure of medical and psychiatric facilities, reduction of CAM-related educational programs at the existing medical schools—, it also reflected broader social and political trends, such as an increasing utilitarianism in American society, the necessity to economize social subsidiaries in the health care system, and the strengthening of the performance of science and medicine in the USA for applications in industry, the agricultural sector, and the military. Quite intriguing in this regard is a comparison with the situation in Germany, Austria, and the Netherlands, which did not experience such strong antagonisms and forms of social regulation as the USA and Canada with the Flexner Report [19]. This difference can be explained by referring to the considerable cultural differences in the acceptance of CAM-oriented research, health care, and education between the German- and English-speaking medical and scientific communities [20].

### 3.2. Comparison with the Reorganization of the CAM Field in Europe from 1960s to 1980s

Professors and chairs of the “1968 generation”—on the academic level—had introduced very different interests (such as research, teaching, and political aims) into university-based medicine in the following two decades, which were often founded on traditional “holistic ideals” (such as psychosomatic medicine, plurality of therapeutic methods, or the broadening of the curative dimension to disease prevention on larger societal scales) [21]. Among current themes, themes, “1968ers” featured an explicit critique of the somatic and organ concentration of the scientific paradigm in medicine as it had originated in the 19th century (among many medical students of the 1970s, the contemporary catchword for example was: “My first patient at medical school was a dead body”), leading to the creation of communication groups for the recording of medical history and for the breaking of bad news (“On the way to communicative medicine”); homoeopathy circles and discussion groups on Complementary and Alternative Medicine (“Nature, not Chemistry”) [22]; political discussion circles on the role of medicine in the global community (such as in local chapters of the “International Physicians for the Prevention of a Nuclear War” and the “*Médecins sans frontières*”); psychosocial psychiatry groups [23]. All of these developments shared a profound criticism of scientific reductionism in medicine, which had gained so much ground since the advent of medical modernity and was also made responsible for many digressions and atrocities of research with human patients in medicine and health care in the 19th and 20th centuries [24, 25].

From the perspective of modern medicine, it had become necessary to understand and control bodily phenomena—and for the sake of argument one would need to abstract from the recent approaches in CAM—[27], clinical thinking, and scientific practices in functional frameworks. At the same time, modern medicine had barely found ways of receiving nonreductionist views in both the medical and psychiatric clinical communities, probably with the exception of psychosomatic physicians, psychoanalysts, and behavioural therapists, who continued to be involved in philosophical considerations about the status of their theories and changes in their practice as a response to the organ-centred and scientific paradigm in medicine and (biological) psychiatry.

In his introductory lectures to psychosomatics, Gerhard Danzer (b. 1956) of the Charité Medical School in Berlin, during the 1990s, particularly emphasized the roots of modern psychosomatics in late 19th and early 20th century publications of the Baden-Baden psychoanalytical physician Georg Groddeck (1866–1934) [28]. Groddeck's supervisor at the University of Berlin, Ernst Schweninger (1850–1924), who was the personal physician of the German Reich's Chancellor Otto von Bismarck (1815–1889), did not prioritize one medical system over any other. He rather developed a holistic approach integrated with elements of deep psychology, psychoanalysis, psychiatry, narrative literature, and physical therapy, which he argued that would avoid the theoretical and practical pitfalls and limitations that 19th century experimental physiology had introduced into contemporary medicine. As in the case of Danzer, the critical works of the foundations of medical science and practice by Georg Groddeck also stimulated a larger group, particularly of German-speaking émigré-psychosomatic physicians in Britain and North America, to focus on additional CAM methods in both the practice of internal medicine and clinical psychiatry [29, 30]. Through the process of forced migration many, leading psychosomatic psychiatrists in the 1930s, such as Franz Alexander (1891–1964) from Budapest and Karl Stern (1906–1975) from Berlin, also introduced Schweniger's and Groddeck's concepts in the American and Canadian psychiatric communities [31]. In particular, psychiatric milieu therapy has advocated for this type of psychotherapy model, by focusing on the total environment in the treatment of mental and behavioral disorders or maladjustments by making substantial changes in a patient's immediate life circumstances, as this was historically advocated for and integrated into the therapeutic approaches of the American child psychiatrist Emmy Sylvester (b. 1910) and the Austro-American psychoanalyst Bruno Bettelheim (1903–1990). A further integration of early CAM approaches with psychiatry was achieved through the advocacy of mind-body-medicine precursors like the Chicago-based psychiatrist and the psychologist Edmund Jacobson (1888–1983) with his introduction of progessive muscle relaxation (PMR) therapy in the 1930s and 1940s (“You must relax”) for mood and anxiety disorders as well as depression [32].

However, such an integration of holistic and psychosomatic approaches with CAM remained the exception rather than the rule until the 1990s, since traditional medical departments had scarcely addressed “integrative perspectives” on “the healing experience” in Central Europe and North America [33]. In this respect, some intriguing comments by German historian of medicine and physiology, Karl Eduard Rothschuh (1908–1984) should be allowed here, when he asked the question “What is and to what end does one study historical medicine?” in a lecture at the Westphalian Wilhelms University of Muenster in 1980 [34]. The lecture plastically summarized the incomplete picture of modern education in “physicianship,” as taught by many medical faculties in the western world, *vis-à-vis* the fragmented body of medical knowledge founded on training in anatomy, physiology, and biochemistry:

*The large weight, which is undoubtedly placed on the natural sciences with regard to the pursuit of medicine's healing tasks, does not mean, however, that medicine itself would be a natural science. Medicine is neither a natural science, nor a humanist discipline. Medicine is not a scientific discipline at all, but is based on scientific disciplines. [...] [Medical History, in addition,] develops and represents a set of values; without Medicina Historica this set of values would not at all be introduced into the medical canon. (The original text can be found in: Rothschuh, 1986 *[34]).**

### 3.3. Impact of the Social Movements of the 1960s and the Opening of the NIH in the US

Differences in philosophical views about the scientific paradigm in medicine, medical reductionism, the place of the patient, and diverging interpretations of medical holism led to intense disputes between physicians, psychiatrists, and alternative practitioners [35]. A time of change had been brought about with the rise of the 1960s, increasing the uses of CAM and widespread discussions about the practice and role of medicine and psychiatry in Western societies and cultures, as is intriguingly represented in the influential criticisms of the Austrian philosopher, theologian, and social scientist Ivan Illich (1926–2002):

*Physical sickness is confined to the body, and it lies in an anatomical, physiological, and genetic context. The “real” existence of these conditions can be confirmed by measurement and experiment, without any reference to a value-system. None of this applies to mental sickness: its status as a “sickness” depends entirely on psychiatric judgment. The psychiatrist acts as the agent of a social, ethical, and political milieu. Measurements and experiments on these “mental” conditions can be conducted only within an ideological framework which derives its consistency from the general social prejudice of the psychiatrist. The prevalence of sickness is blamed on life in an alienated society, but while political reconstruction might eliminate much psychic sickness, it would merely provide better and more equitable technical treatment for those who are physically ill. (The original text can be found in: Illich, 1976) [36].*

Of course, these criticisms of the scientific paradigm in medicine were by no means a homogenous trend, but rather triggered through a heterogeneous mixture of social, medical, and psychiatric movements, events, and developments that impacted the changes towards auxiliary and increased use of Complementary and Alternative Medicine and Psychiatry in places where modern medicine had little if nothing to offer (e.g., chronic pain management, oncology and palliative care, therapy of complex psychiatric disorders with compliance problems, etc.).

The “hippie movement”—on the broader level of society—was certainly one important strand among these heterogenous criticisms, in which virtues of a simple, natural life, tolerance of diverse lifestyles, consumption of natural and organic foods, and the social use of psychoactive drugs were promoted [37]. Also, the human potential movement is worth mentioning as they advocated for therapeutic approaches such as vegetarianism, natural birthing, transcendental meditation, yoga, and biofeedback. Its participants were concerned with the quality of both personal life and social life in the modern world, such as the preceding protagonists of psychosomatic medicine, wellness, movement, and humanistic medicine. In North America, this movement was centered around the foundation of the Academy of Psychoanalytic Medicine (APM), in 1954, and the address by Halpert L. Dunn (1896–1975) from the US Public Health Service on the concept of wellness in the early 1960s, which broke with earlier disease-based models that had developed during the 19th century scientific paradigm of medicine. Dunn introduced a new integrated concept of health and wellbeing, “which is oriented toward maximizing the potential of which the individual is capable, within the environment where he is functioning” [38].

Socially, the tradition of postmodernism, feminism and environmentalism were also crucial for the reaction to the previous era of modernism, characterized by the belief in the existence of truth, objectivity, determinacy, causality and impartial observation and with an emphasis on individuality, complexity, and personal experience. These changes became further integrated into the social construction of curricula and values in the medical system in the 20th century [39].

The development of Complementary and Alternative Medicine and Psychiatry after the publication of Flexner's 1910 Report to the American Carnegie Foundation was manifold and in certain respects was also fruitful. On the one hand, Flexner's work led to the closure of colleges, hospitals, and programs in which “unconscionable quacks” were working who had been “a disgrace to the State,” as the author of the report wrote. The political and disciplinary crackdown on alternative and nonconventional forms of research and education in medicine and psychiatry, on the other hand, did not reach the general population, nor did its beliefs about the doctor-patient relationship and other forms of healing and medical support. Largely due to such outside developments, plans for integrative forms of medical practice that selectively incorporated elements of CAM evolved into comprehensive treatment plans alongside solidly orthodox methods of diagnosis and health care [40]:

*Integrative Medicine is the practice of medicine that reaffirms the importance of the relationship between practitioner and patient, focuses on the whole person, is informed by evidence, and makes use of all appropriate therapeutic approaches, healthcare professionals and disciplines to achieve optimal health and healing. (Consortium of Academic Health Centers for Integrated Medicine) [41].*

However, in many ways the current status of “integrative medicine” (IM) in medical and psychiatric institutions in North America is still (and importantly) future oriented in its thinking—although problematic in vision—, as relatively few schools have really integrated conventional medicine with Complementary and Alternative Medicine and Psychiatry, at least not until recently.

### 3.4. The Recent Chronology—Increasing International CAM and IM Platforms

The creation of the National Center for Complementary and Alternative Medicine (NCCAM) in the USA in 1991—even though it was given only a comparatively small budget below one percent of NIH's expenditures at the time, brought about by the US Senator Tom Harkin (b. 1939), proved to be a landmark event in the renewed support of CAM in North America [42]. Large scale research could now be pursued under the leadership of the NIH, by combining mainstream medical therapies and CAM approaches, while investigating scientific evidence, safety, and efficacy:

*CAM is a group of diverse medical and health care systems, practices, and products that are not presently considered to be part of conventional medicine. Conventional medicine is medicine as practiced by holders of M.D. (medical doctor) or D.O. (doctor of osteopathy) degrees and by their allied health professionals, such as physical therapists, psychologists, and registered nurses. Some health care providers practice both CAM and conventional medicine. While some scientific evidence exists regarding some CAM therapies, for most there are key questions that are yet to be answered through well-designed scientific studies—questions such as whether these therapies are safe and whether they work for the diseases or medical conditions for which they are used. The list of what is considered to be CAM changes continually, as those therapies that are proven to be safe and effective become adopted into conventional health care and as new approaches to health care emerge. (NCCAM, 2007) [43].*

In its inaugural year of 2004, the international Consortium of Academic Health Centers for Integrated Medicine (CAHCIM) expressed the future hope that integrative medicine would become the cornerstone of the urgently needed reconstruction of what was perceived as a dysfunctional healthcare system, including both the somatic and the psychiatric fields. The development of new frameworks of CAM has also realized that genetic and translational aspects of modern biomedical and psychiatric research had their place in such a new health care paradigm [44]. In fact, psychiatrists and psychologists today display an increasing intellectual openness towards the use of CAM and integrative approaches in their therapeutic practice, as well as the growing evidence base for specific CAM modalities in both the treatment and prevention of mental illness and disease [45]. These major changes have largely occurred in the USA, or in countries that have adopted the US model. While the federal organization of Health Canada has also played an important role in Canada, quantitatively there has been only a minor level of involvement, especially when compared to the USA.

With the creation of the International Network of Integrative Mental Health (INIMH) in 2010, there now exists an important institutional platform which furthers the development of a biopsychosocio-spiritual model in integrative mental health that is evidence based. Some of these promising changes, which are taking place in mental health care in Western countries, are represented, for example, in the growing use of homoeopathy in mood and mild anxiety disorders and the increasing role of traditional Chinese acupuncture therapies in the management of chronic pain conditions, depression, and anxiety disorders, as well as folate and other substitutional nutritional factors in depression and bipolar disorders [46].

## 4. Conclusions

**Figure 5:**
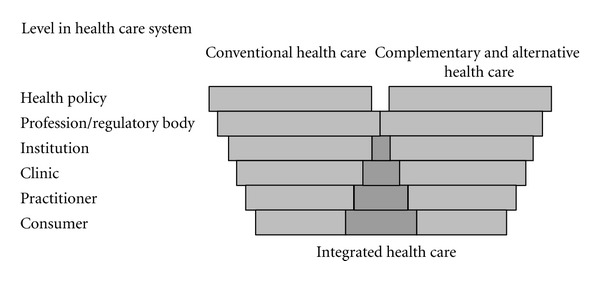
Level in health care systems [26].

For some, the real trend in CAM medicine and psychiatry has become evidence-based medicine (EBM), not complementary and alternative medicine itself. This observation further aligned with the fact that medical, and increasingly also psychiatric education, has changed considerably over the past decade along with new trends in CAM education [47], while EBM is now infiltrating medical school curricula on both the basic science and clinical care ends [48]. While this paper has looked back from a history of medicine perspective at the publication of the Flexner Report one century ago, it should also be emphasized that the Flexner Report was revolutionary and and is even today even today widely celebrated as a seminal document that subsequently raised the standards for general education in medicine and psychiatry. However, the last decades have also seen a disturbing trend away from Flexner's prescriptions, since medical schools are reverting to many of the pre-Flexnerian standards by uncritically adding many pseudoscientific health claims to their course materials as “IM,” without rigorous tests, studying CAM practice or asking trained physicians for their experiences (Figure 5). Certainly, Flexner himself would have “approved” of a new evidence-oriented direction in medical and psychiatric education:

*Unfortunately, Flexner may be rotating all too rapidly. […] medical schools are teaching and promoting what is often called CAM, despite the lack of logic or evidence supporting many CAM practices. Meanwhile, the same schools seem to give only lip service to the application of logic and evidence to healthcare, as exemplified by the formal processes of EBM. [49].*

We increasingly recognize today that treatment is not an isolated event in patients' lives, but it takes part in the patient's own bio-psycho-social context, which includes social networks, patients' subjective experiences, and their mental health status, along with the patient provider relationship (a system). These elements are crucial to testing an intervention, as a patient is not an average patient, with average beliefs, devoid from any contextual influences [50]. As CAM treatments in psychiatry become more and more efficient and safe, as well as increasingly supported by data from randomized controlled trials and other EBM methodologies in clinical epidemiology, new standards for an appropriate and reliable use of complementary and alternative medicine and psychiatry are emerging, which go hand in hand with recommendations for the monitored and evaluated use of CAM and integrative therapies in mental health care in the USA, Canada, and other developed countries [51].

In this context, INIMH aims at augmenting and adapting approaches in contemporary psychiatry, along with biomedical perspectives in health care and research and its attempts to work out a more adequate paradigm, one of which aims at transcending the boundaries of what Abraham Flexner had laid out a century ago in his influential Report on *Medical Education in the United States and Canada* [52].
